# Generation of Human Female Reproductive Tract Epithelium from Human Embryonic Stem Cells

**DOI:** 10.1371/journal.pone.0021136

**Published:** 2011-06-15

**Authors:** Louie Ye, Robyn Mayberry, Camden Y. Lo, Kara L. Britt, Edouard G. Stanley, Andrew G. Elefanty, Caroline E. Gargett

**Affiliations:** 1 The Ritchie Centre, Monash Institute of Medical Research and Department of Obstetrics and Gynecology, Monash University, Melbourne, Australia; 2 Monash Immunology and Stem Cell Laboratories, Monash University, Melbourne, Australia; 3 Monash Micro Imaging, Monash University, Melbourne, Australia; 4 Anatomy and Developmental Biology, Monash University, Melbourne, Australia; The University of Hong Kong, Hong Kong

## Abstract

**Background:**

Recent studies have identified stem/progenitor cells in human and mouse uterine epithelium, which are postulated to be responsible for tissue regeneration and proliferative disorders of human endometrium. These progenitor cells are thought to be derived from Müllerian duct (MD), the primordial female reproductive tract (FRT).

**Methodology/Principal Findings:**

We have developed a model of human reproductive tract development in which inductive neonatal mouse uterine mesenchyme (nMUM) is recombined with green fluorescent protein (GFP)-tagged human embryonic stem cells (hESCs); GFP-hESC (ENVY). We demonstrate for the first time that hESCs can be differentiated into cells with a human FRT epithelial cell phenotype. hESC derived FRT epithelial cells emerged from cultures containing *MIXL1^+^* mesendodermal precursors, paralleling events occurring during normal organogenesis. Following transplantation, nMUM treated embryoid bodies (EBs) generated epithelial structures with a typical MD phenotype that expressed the MD markers PAX2, HOXA10. Functionally, the hESCs derived FRT epithelium responded to exogenous estrogen by proliferating and secreting uterine-specific glycodelin A (GdA).

**Conclusions/Significance:**

These data show nMUM can induce differentiation of hESC to form the FRT epithelium. This may provide a model to study early developmental events of the human FRT.

## Introduction

During embryogenesis, the mesoderm emerges from the primitive streak and gives rise to coelomic epithelium. The Müllerian Duct (MD) arises from invagination of coelomic epithelium during fetal development. Subsequently, the MD gives rise to the human female reproductive tract (FRT) that further differentiates to form the oviduct, uterus and upper vaginal canal. The mucosal lining of the uterus is known for its remarkable regenerative capacity during a female's reproductive years. Recently, the regenerative capacity of the endometrium has been attributed to a small population of resident stem/progenitor cells. Our laboratory discovered these cells in both the stroma and epithelium of the adult human and murine uterus [1], [2], [3]. We have identified cell surface markers that enrich for endometrial mesenchymal/stromal stem/progenitor cells, and ongoing investigations now focus on finding definitive markers for the epithelial stem/progenitor cells. Identifying and characterising these stem/progenitor cells will provide a better understanding of the normal cyclical regenerative processes in human endometrium and the pathophysiology of human endometrial proliferative diseases, such as endometriosis, endometrial hyperplasia and endometrial cancer.

Recent studies have shown that creating developmental models from embryonic stem cells (ESCs) is a tractable approach to track and potentially identify adult stem/progenitor cells [4], [5]. In this context, we believe a hESC based model of human MD development will facilitate the identification and characterization of female reproductive tract stem/progenitor cells. Tissue recombination is a powerful tool for studying stromal-epithelial interactions. For example, neonatal mouse uterine mesenchyme (nMUM) had been recombined with human and mouse uterine epithelial cells in previous tissue recombination experiments [6], [7], [8], [9]. nMUM also transdifferentiates pluripotent spermatogonial stem cells into murine uterine epithelial tissue [10] and a number of studies have shown that specific stromal populations can direct ESC differentiation towards derivatives of their corresponding epithelia, including bladder, prostate and oocytes [4], [5], [11]. We hypothesized that nMUM might provide inductive cues capable of directing hESCs to differentiate into human FRT epithelium. We adopted established methods for hESC differentiation to form embryoid bodies (EB) from green fluorescent protein-tagged hESCs; GFP-hESCs (ENVY). EBs were then combined with nMUM and the resultant recombinant subsequentlty grafted into immunocompromised mice. We demonstrated that nMUM induced differentiation of hESCs to form human FRT epithelium in a process that paralleled known stages of human FRT organogenesis.

## Results

### Neonatal mouse uterine mesenchyme directed hESCs to form human female reproductive tract epithelium in vivo

**Figure 1:**
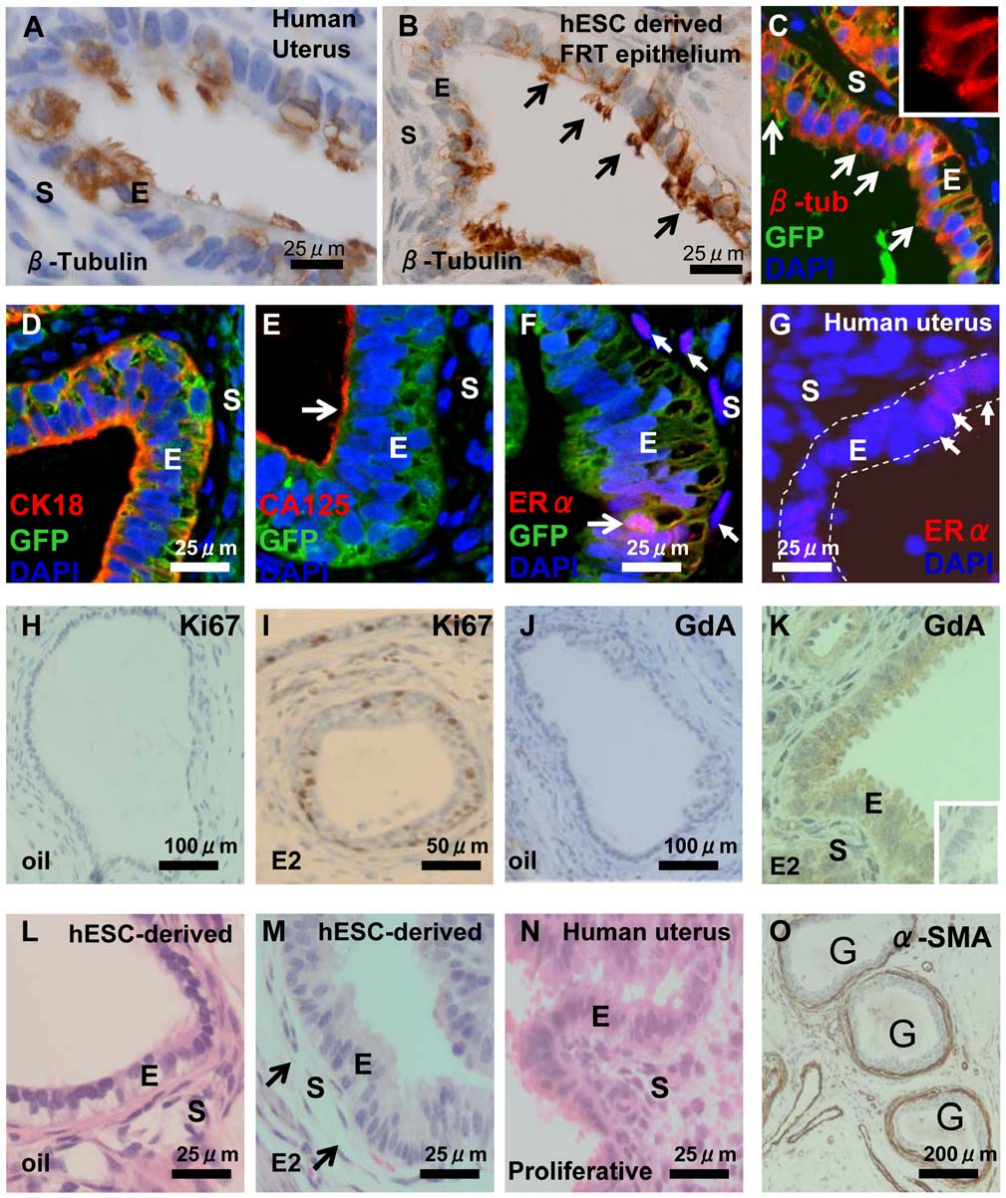
Characterisation of hESC-derived FRT epithelium in GF treated heterotypic (nMUM) recombinant 8 week xenograft. β-tubulin expression in (**A**) ciliated simple columnar epithelia of human adult proliferative endometrium and (**B**) in hESC derived ciliated, simple columnar FRT epithelium (arrows indicates cilia). (**C**) immunofluorescence analysis of hESC derived FRT epithelium (green) where β-tubulin (yellow orange) is expressed on cell surface (inset shows a close up view of the cilia), the hESC origin of epithelial cells was assigned on the basis of GFP expression. Note that adjacent mouse uterine stromal cells are GFP**^−^**. hESC derived FRT epithelium co-localised (**D**) cytoplasmic CK18, (**E**) CA125 on epithelial surface (arrow), and (**F**) ERα (pink) with GFP^+^ hESC derived epithelial cells. Weak ERα expression was also evident in mouse uterine stromal cells (filled arrows). (**G**) ERα expression in human proliferative endometrial gland (full arrows), dotted line indicates epithelium. Ki67 expression in hESC derived FRT epithelium before (**H**) and after (**I**) estrogen injections showing estrogen-induced epithelial cell proliferation. The expression of GdA in hESC derived FRT epithelium without (**J**) and with (**K**) estrogen injections showing estrogen-induced cytoplasmic expression of GdA. H&E of hESC derived FRT epithelium lined by simple columnar cells without (**L**) and with (**M**) estrogen treatment, showing estrogen-induced increase in epithelial height and stromal oedema (arrows), classical hormonal responses of adult uterine epithelium (**N**). (**O**) Three hESC derived FRT epithelial structures surrounded by α-SMA positive cells. Inset in (**K**) is concentration matched mouse IgG1 negative control. Hoechst stained serial sections corresponding to (**I**) and (**K**) are found in Figure S6E–F. Abbreviations: α-SMA, α-smooth muscle actin; CA125, cancer antigen 125; E, epithelium; ERα, estrogen receptor alpha; E2, estrogen; G, gland; GdA, glycodelin A; S, stroma.

No single marker defines the adult FRT epithelium which includes the oviduct, uterus and upper vaginal canal. Therefore we used a previously established combination of a morphological marker (cilia) and the immunohistochemical markers cancer antigen 125 (CA125), Glycodelin A (GdA) and estrogen receptor alpha (ER-α) to identify the FRT epithelium [12], [13]. As a prelude to experiments utilising nMUM mesenchyme, we first tested the ability of differentiating hESCs to spontaneously differentiate into human FRT epithelium following transplantation. To this end, we grafted ovariectomized mice with two types of controls; EBs formed in the absence of growth factors (n = 4) or EBs treated with BMP4/ACTIVIN A (n = 4), growth factors known to induce hESCs to differentiate towards mesendoderm, an obligate intermediate during FRT development. Lack of ovarian hormones does not impair ciliogenesis or development of the female reproductive tract [14], [15], [16], but the absence of these hormones enables distinction of reproductive and respiratory tract epithelia. Specifically, under these conditions, normal human uterine epithelium is a ciliated simple columnar epithelium (Figure 1A), in contrast, respiratory epithelium is ciliated and pseudostratified (data not shown). We found no evidence of ciliated simple columnar epithelium in either control group, however, we detected ciliated pseudostratified columnar epithelium resembling respiratory epithelium similar to that described in a previous report [17]. In addition to these hESC controls, we also grafted nMUM alone and found no evidence of epithelial differentiation, consistent with previous studies (data not shown) [7], [10]. In the experimental groups, we detected ciliated simple columnar epithelium in each graft derived from both the non-growth factor treated (n = 2) (data not shown) and growth factor treated EB/nMUM recombinant tissues (n = 30) (Figure 1B, C). Grafts derived from these samples were smaller than those arising from EBs formed in the absence of nMUM and/or growth factors, (Figure S1A), consistent with previous studies showing that differentiated hESC had reduced the potential for teratoma formation [5], [17]. We also found the viability of hESC differed between immunodeficient host mouse strains and that NOD.Scid gamma (NSG) mice were ideal for hESC cell development compared to NOD.Scid mice (data not shown) consistent with a previous study [18].

In order to confirm that ciliated structures were indeed hESC derived epithelium, we used a combination of Hoechst stain and anti-GFP antibody staining (Figure 1C–F, Figure S6A). This analysis enabled GFP^+^ ENVY-derived human epithelial cells containing smooth Hoechst-stained nuclei to be distinguished from GFP^−^ mouse cells with speckled nuclei [19]. In week 8 recombinant grafts, ciliated simple columnar epithelium was only observed when surrounded by mouse stromal cells (Figure 1C). In GF-treated week 8 recombinant grafts, we found epithelial tissue made up a small percentage of the total area (4.8±2.3%), and that ciliated simple columnar human epithelium accounted for 33.8±22.6% of all epithelial structures (n = 4, mean±s.e.m.). Consistent with a previous report, our data showed that a considerable portion of each graft consisted of non-epithelial tissue and fluid filled cavities (Figure S1C) [17]. In addition, the proportion of ciliated simple columnar epithelial structures in recombinant grafts varied; for instance, in one graft, the only epithelial structure present was ciliated whilst the rest of the graft consisted of fluid-filled cavities (Figure S1C). In other grafts, several hESC derived FRT epithelial structures were present (Figure S1B, Figure S1D). Despite this variability, structures with characteristics consistent with FRT epithelium were found in all grafts where both nMUM and hESC derived cell types were present (n = 30) (Figure S2).

Immunofluorescence analysis indicated that the hESC derived FRT epithelium expressed human CK18 and CA125 (Figure 1D, Figure 1E). This result excludes the possibility that these structures represented luminal epithelium of the male reproductive tract or ependymal epithelium of the central nervous system, which do not express CA125 [20]. Following exogenous estrogen treatment, estrogen receptor-α (ERα) was detected in the hESC derived FRT epithelium (Figure 1F, Figure S4A) albeit at a low frequency and variable intensity, comparable to ERα expression in human endometrial glands (Figure 1G, Figure S4C, Figure S4D). In recombinant grafts, ERα is only detected in mouse stromal cells and hESC derived FRT epithelium (Figure S4A, Figure S4B). To assess the functional capacity of the hESC derived FRT epithelium, we treated host mice with multiple estrogen injections over 5 days [9]. Estrogen induced epithelial proliferation, as demonstrated by Ki67 expression (Figure 1I), similarly, the hESC derived epithelia in estrogen treated animals expressed GdA in the cytoplasm (Figure 1K). We also observed morphological changes in response to exogenous hormonal stimulation, the hESC derived FRT epithelium increased in height, whilst the underlying stromal cells demonstrated edematous change (Figure 1M), mirroring changes that occur in normal cycling adult proliferative uterine epithelium and stroma (Figure 1N). In contrast, grafts from vehicle alone treated hosts contained low cuboidal epithelium and lacked stromal edema (Figure 1L). Furthermore evidence of reciprocal interaction between differentiated human epithelial cells and mouse endometrial stromal cells was indicated by the expression of alpha - smooth muscle actin (α-SMA) in the latter (Figure 1O), consistent with previous studies [4], [5]. Collectively, these results demonstrate that nMUM can direct hESCs to differentiate into hormonally responsive FRT epithelium.

### FRT epithelia arises from MIXL^+^ embryoid bodies

**Figure 2:**
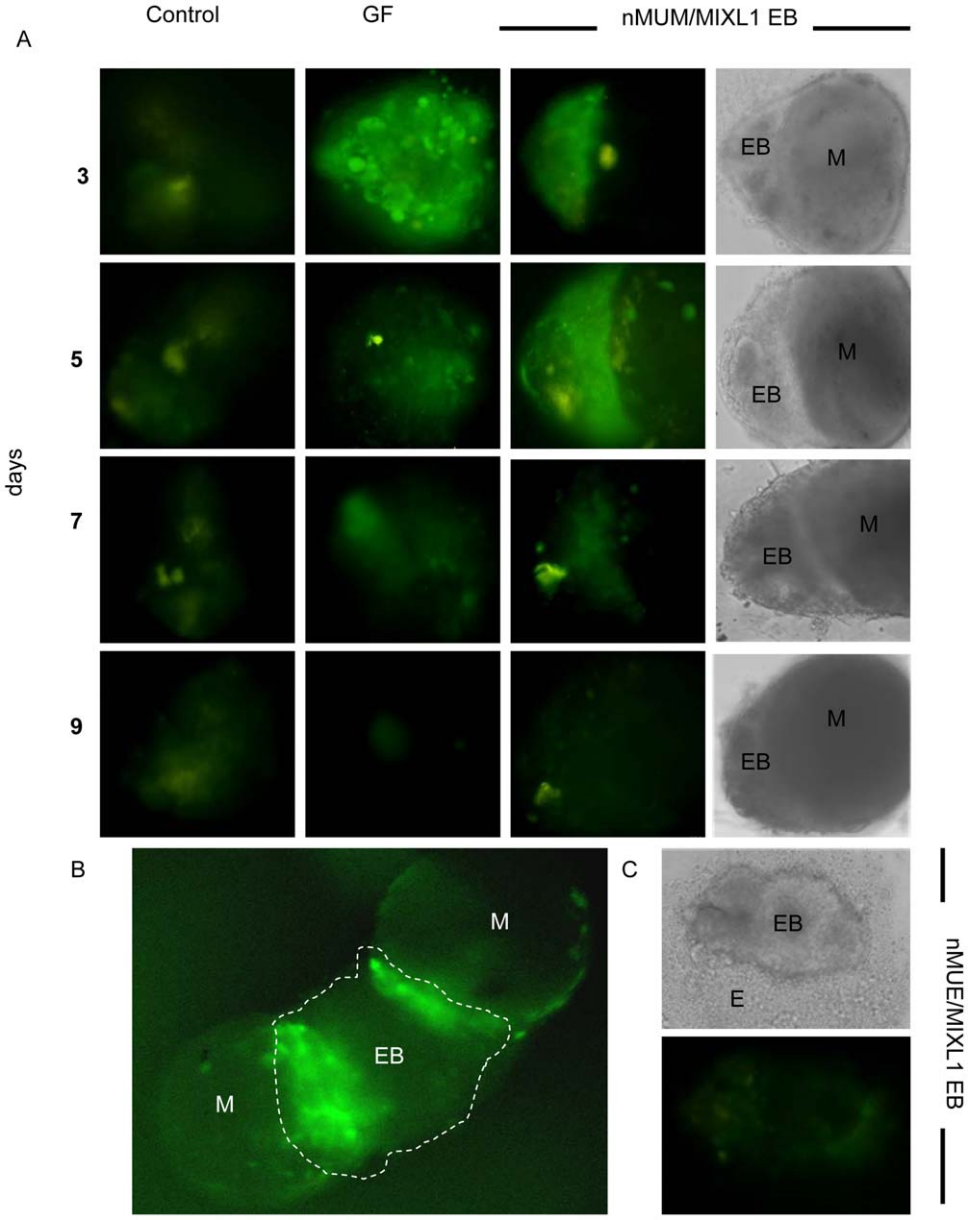
Neonatal mouse uterine mesenchyme induces MIXL1 expression in MIXL1^GFP/w^ EBs. (**A**) nMUM induced GFP expression in *MIXL1* ^GFP/w^ EB recombinants after three days of co-culture (3^rd^ panel), representative example of n>100. The duration of reporter activation is comparable to that observed in growth factor treated EBs (2^nd^ panel, days 3,5), while little to no reporter activity was detected in the control (1^st^ panel) (n>100) (×40 magnification, first 3 panels are fluorescent images, images in 4^th^ panel represent phase contrast images of recombinants in 3^rd^ panel). (**B**) Co-culture of two nMUM pieces (0.5 mm) with a larger EB (>4000 cells) activated reporter activity locally (dotted area is the EB) (×20 magnification). (**C**) No reporter activity was detected in embryoid bodies co-cultured with neonatal uterine epithelium (0.5 mm) (×40 magnification). Areas marked as mesenchyme in recombinants are based on morphological evidence and recombination experiments using ENVY hESC (refer to Figure S2A–C). Abbreviations: EB, embryoid body; E, neonatal mouse uterine epithelium; GF, growth factors; M, neonatal mouse uterine mesenchyme.

Since MD is derived from primitive streak mesendoderm, we sought to determine if nMUM induced formation of mesendodermal cells in differentiating human EBs. To examine this process, we generated EBs with a *MIXL1^GFP/w^* hESC reporter line in which GFP expression has been placed under the control of the of the primitive streak gene, *MIXL1*. We detected GFP expression as early as day 3 in developing EBs cultured in serum-free-medium in the presence of nMUM (Figure 2A). Reporter activity in the recombinant EBs, which paralleled that seen in EBs treated with growth factors (BMP4 and ACTIVIN A), first appeared on day 3 and gradually diminished by day 7 (Figure 2A). Furthermore, in larger EBs (>4000 cells), reporter activity was localised to the area of contact between EB and mesenchyme (Figure 2B, Figure S3). When we recombined *MIXL1^GFP/w^* EBs with neonatal mouse uterine epithelial (nMUE) cells under serum free conditions, we observed no reporter activity (Figure 2C). These observations suggest close range morphogens from nMUM, but not nMUE, directed the differentiation of hESC to mesendodermal cells in *MIXL1^GFP/w^* EBs. These observations indicate that nMUM, but not nMUE, produced factors capable of directing the differentiation of hESCs to mesendodermal progenitors.

### Expression of mesoderm markers in EBs induced to differentiate with nMUM

**Figure 3:**
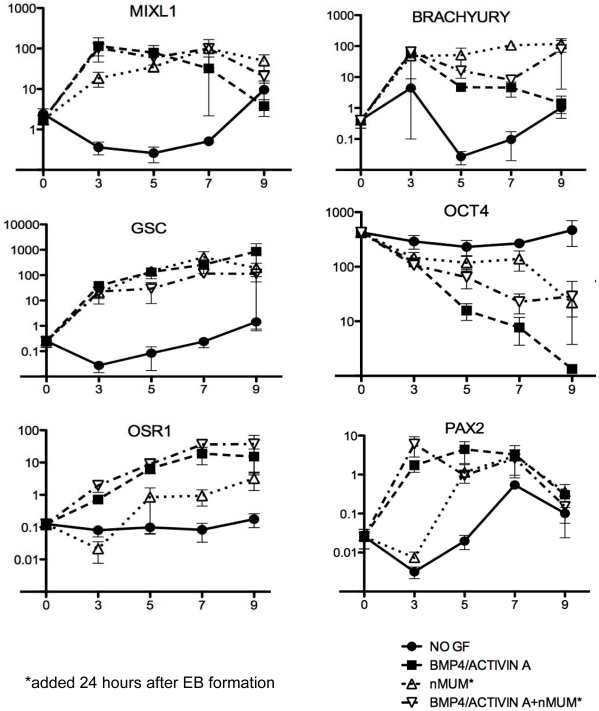
Neonatal mouse uterine mesenchyme induces expression of primary germ layer markers in ENVY/nMUM recombinants. Real time PCR analysis of RNA collected from EB and recombinants cultured in serum-free BPEL medium. Growth factors (BMP4, 50 ng.ml^−1^ and ACTIVIN A, 20 ng.ml^−1^) were added immediately after forced aggregation of hESCs. Neonatal mouse uterine mesenchyme was added 24 hours after EB formation into either growth factor treated or untreated EB culture. Expression of genes relative to GAPDH were analysed by quantitative RT-PCR after 3, 5, 7 and 9 days incubation. Expression of target genes in undifferentiated hESCs is indicated as day 0 of differentiation (Data is plotted as mean±s.e.m., n = 3 independent differentiation experiments). Abbreviations: GF, growth factor; nMUM, neonatal mouse uterine mesenchyme.

We hypothesized that nMUM might induce the differentiation of *MIXL1^+^* mesendodermal progenitors to mesoderm with characteristics of lateral plate mesoderm. In the first instance, we analysed the expression of genes which mark cells from the mesendoderm and mesoderm in the differentiating recombinant ENVY EBs from day 3 to day 9 using qRT-PCR. In the presence of growth factors (BMP4 and ACTIVIN A) alone, *MIXL1*, *Brachyury (T)*, *PAX2*, were rapidly upregulated and slowly declined over time (Figure 3). *Goosecoid (GSC)*, and *OSR1* increased over time compared to control (no growth factor). High levels of *MIXL1*, *GSC*, and *T* suggest differentiation of hESCs towards anterior population of mesendodermal cells, consistent with the role of ACTIVIN A [21], [22]. In addition, we also observed the upregulation of intermediate and lateral plate mesodermal markers *OSR1* and *PAX2*, consistent with a previous study, suggesting that under these conditions, EBs potentially harbour a precursor of MD [23]. nMUM upregulated *MIXL1*, *T*, and *GSC* as early as day 3, but there was delayed upregulation of *OSR1* compared to growth factor treated EBs (Figure 3). *PAX2* showed little change compared to control over time. Gene expression profiles of recombinant EBs treated with growth factors were similar to that of EBs treated with growth factor alone, although the nMUM appeared to maintain higher levels of expression for *MIXL1, and T*, on day 9 compared to GF treated EBs (Figure 3). The pluripotency marker Oct4 decreased steadily over time in all groups except control. These results suggest that in the absence of growth factors, nMUM can promote hESC differentiation towards mesendoderm, albeit less efficiently than BMP4/ACTIVIN A. However, despite the similar gene expression profile of growth factor only treated EBs and growth factor treated EB/nMUM recombinants, the former never developed FRT epithelium following transplantation.

### nMUM induced differentiation of hESCs recapitulates aspects of rodent and human Müllerian Duct development

**Figure 4:**
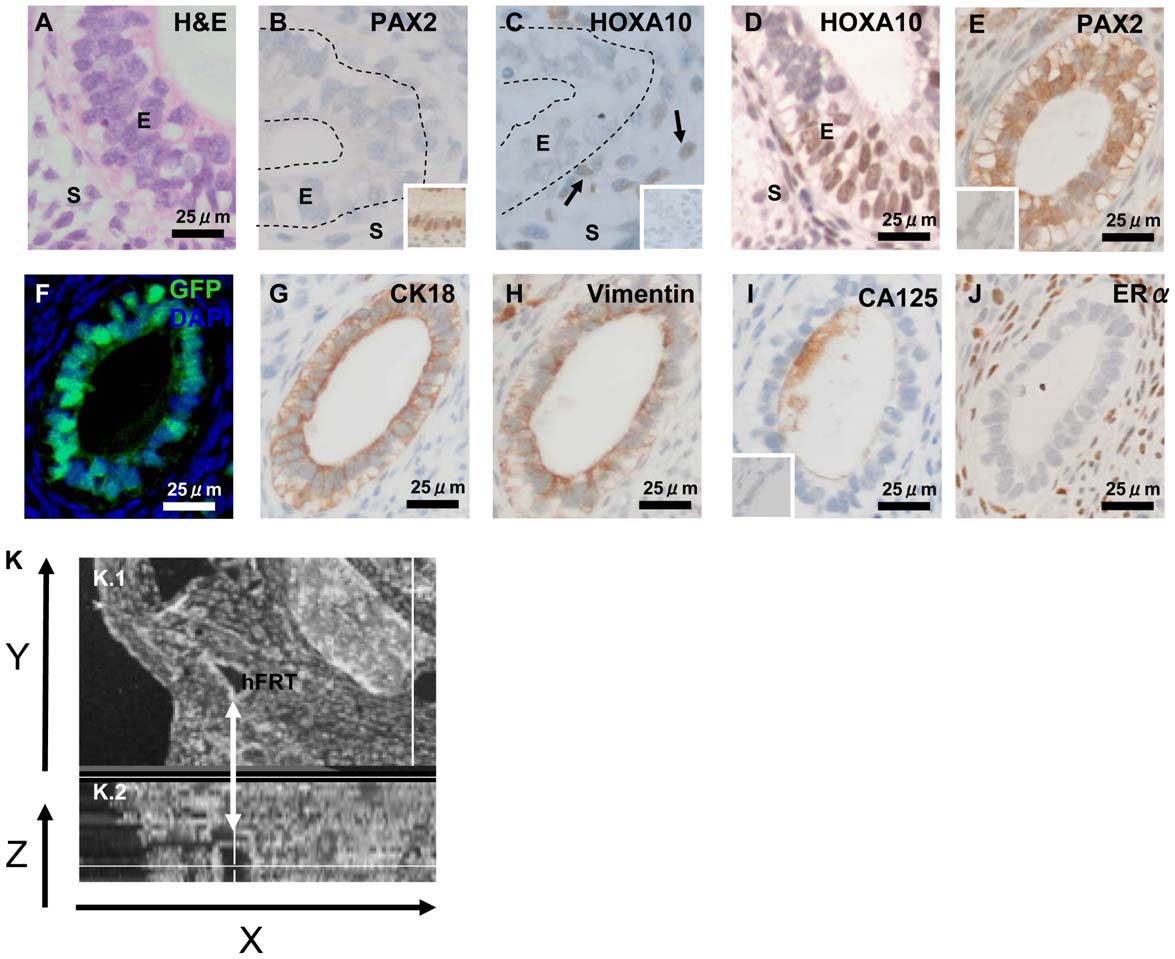
Morphological and immunohistochemical characteristics of hESC derived FRT epithelial structures in grafted GF treated recombinants. (**A**) H&E showing pseudostratification in the hESC derived FRT epithelium surrounded by co-transplanted mouse stroma in a 4 week graft. (**B**) no *PAX2* or *HOXA10* (**C**) expression in week 2 hESC derived FRT epithelium (**B, C**) were serial sections representative of two week 2 graft) (arrows point to *HOXA10^+^* mouse stromal cells). (**D–I**) Representative sections from two different 4 week grafts, (**E–J**) serial sections of a single gland (sectioned at 3 µm), (**D, E**) Nuclear expression of *HOXA10* and *PAX2* in hESC derived FRT epithelium at 4 weeks. (**F**) representative serial sections of the same gland depicted from (**E–J**) showing GFP^+^ hESC derived epithelium surrounded by GFP^−^ mouse uterine stromal cells. The gland co-localised cytoplasmic expression of (**G**) CK18 and (**H**) Vimentin in hESC derived FRT epithelium. (**I**) CA125 expressed on hESC derived FRT epithelium surface. (**J**) ERα expressed in transplanted mouse stromal cells but absent from hESC derived FRT epithelium. Insets in (**B**) is a positive control of PAX2 expression in adult human endometrial epithelium, inset in (**C**) concentration matched goat IgG negative control, inset in (**E**) concentration matched rabbit IgG negative control, inset in (**I**) mouse IgG1 negative control for (**G–I**). (**K.1–2**) 3D reconstruction of hESC derived FRT epithelium from serial sections depicted in (**E–J**) (arrow) shown on the Y-axis, (**K.2**) shown on the Z-axis view as a duct. Abbreviations: E, epithelium; hFRT, hESC derived FRT; HOXA10, Homeobox A10; PAX2, Pair box gene 2; S, stroma.

To investigate the kinetics of MD development following EB transplantation, we harvested recombinant grafts at 2 and 4 weeks. hESC-derived FRT epithelial structures were detected at both time points (Figure 4) and comprised of areas of pseudostratification (Figure 4A), as described in a previous report of human MD epithelium [24]. To determine the stage of development, we examined expression of known MD developmental markers such as *PAX2* and *HOXA10* [25]. *PAX2* expression first appears after MD specification, during the elongation stage of development [26]. Although at 2 weeks, *PAX2* and *HOXA10* expression was not detected in the hESC derived FRT epithelium (Figure 4B–C), at 4 weeks, *HOXA10* and *PAX2* expression was observed in the developing hESC derived FRT epithelium (Figure 4D–E, Figure S5A, Figure S5B), consistent with the temporal expression pattern previously reported for the elongation and differentiation stages of MD development in mice [25], [27]. Co-expression of CK18 (Figure 4G) and Vimentin (Figure 4H, Figure S5C) in cells present on serial sections suggested that the hESC derived FRT epithelium was meso-epithelial in character. In addition, the expression of CA125 (Figure 4I, Figure S5D) supports our hypothesis that like human and rodent MD, hESC derived MD originates from a coelomic-like epithelia [12], [28]. The complete absence of ERα in the hESC derived MD epithelium (Figure 4J) at this stage of development is consistent with previous reports showing human fetal MD lacks ERα expression [29]. Motile β-tubulin-expressing cilia were not detected on the hESC-derived FRT epithelium (data not shown). We then optically reconstructed a hESC derived FRT gland from stained serial sections (depicted from Figure 4E–J), and found it was ductal in character (Figure 4K). These data indicate that following in vitro differentiation, nMUM induced MD differentiation from hESCs recapitulated some of the known sequential stages MD ontogeny.

## Discussion

**Figure 5:**
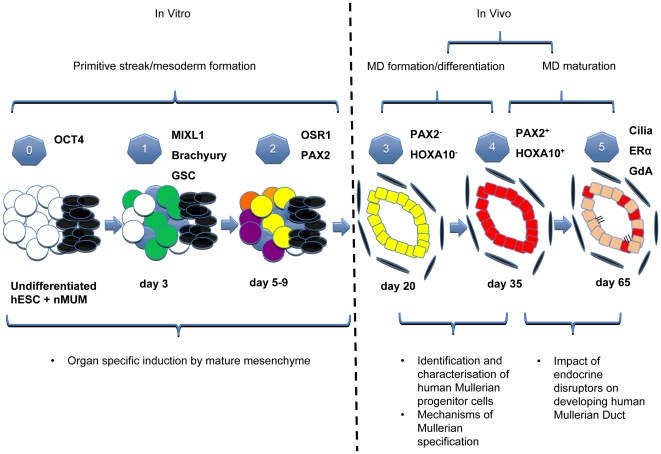
Schematic of the proposed Human Müllerian Duct development model and its potential applications. Stages of development of the hESCs/nMUM recombinant in vitro and in vivo. Key stages are numbered from 0–5 accompanied by some stage specific markers. (**0**): Undifferentiated hESC cells (white circles) were recombined with nMUM (black ovals), (**1**): primitive streak formation: nMUM (black ovals) induced some hESC to differentiate into MIXL1^+^ mesendodermal cells (green circles) whilst the rest remained either as undifferentiated (white circles) or belonging to the ectodermal lineage (blue circles), (**2**): germ layer induction: mesodermal cells (yellow circles) derived from MIXL1^+^ mesendodermal cells in the previous stage, other colored circles represent a variety of differentiated (purple, orange, blue) and undifferentiated cells (white circles), (**3**): in vivo differentiation, and formation of a pre-Müllerian epithelium (yellow) possibly derived from mesodermal cell types in the EB (the presence of tissues from other lineages is omitted from this figure for illustration purposes), (**4**): transition from the pre- Müllerian epithelium into MD epithelium (red), (**5**): maturation of MD epithelium into adult FRT epithelium (pink) containing residual MD epithelial cells (in red). Text boxes (below schematic) include potential applications of the model at different stages of development.

Very few studies have examined the developing human MD [24], [30], [31] and none have examined the differentiation of hESCs to mesoepithelial derivatives. In this study we established a model of MD development, providing an opportunity to investigate factors affecting the genesis of the human FRT. In contrast to recent work where nMUM was used to differentiate spermatogonial stem cells into murine uterine epithelium [10], developmental stages observed in our model are similar to normal Müllerian organogenesis, making it an ideal platform to study human FRT development (Figure 5). Our current protocol involves two distinct stages; hESCs were first differentiated towards cells expressing mesodermal markers in vitro, and then transplanted in vivo to form the hESC derived FRT epithelium expressing markers of the MD and its derivatives. To our knowledge, this is the first report of hESC differentiation into mesodermally derived epithelium, in this case the human FRT epithelium.

In order to identify MD epithelium and its derivatives in the recombinant grafts, we examined a short list of commonly known morphological and immunohistochemical markers of adult human female reproductive (oviduct/uterine) epithelium used in routine diagnostic laboratories and other tissue recombination studies [7], [10]; cilia (β-tubulin), CK18, ERα, CA125 and GdA. Our results indicate only the hESC derived FRT epithelium in our recombinant grafts expressed this suite of epithelial markers. Several estrogen-induced functional responses of FRT epithelium were also demonstrated; increased epithelial proliferation, a defining feature of the uterine epithelium [8], [9], [10], and secretion of GdA. GdA is found in both oviduct (uterine tube) and uterine epithelium of the female reproductive tract and is produced by epithelium in response to estrogen or progesterone [13], [32]. Low levels of ERα expression in the hESC derived FRT epithelium suggests that the action of estrogen on the recombinant structure may be mediated via ERα in the underlying mouse stroma by a paracrine mechanism, recapitulating a characteristic feature of stromal-epithelial interactions in mouse and human reproductive tract tissue [9]. Other evidence of stromal-epithelial interaction is the smooth muscle differentiation of the mouse stromal cells, consistent with previous recombination studies using hESCs and other urogenital tissues [4], [5]. One possible explanation for the weak expression of ERα is that our model developed in the absence of endogenous ovarian hormones. However, since MD development is an estrogen-independent process, the absence of ovarian hormones did not impair formation and differentiation of the MD, as expected [15], [16]. Together these data suggest that nMUM directed differentiation of hESCs to become human FRT epithelium. However, as a recombinant model consisting of mouse and human cells, one would expect some functional differences between our hESC derived FRT epithelium and that of the normal human FRT epithelium especially in the area of hormone responsiveness of the tissue as demonstrated previously in other uterine recombinant models [9]. In future experiments, for further characterisation of the hESC derived FRT epithelium, we plan to compare the molecular signatures between our FRT epithelium with the normal human FRT epithelium.

Animal studies have shown that coelomic epithelium is specified to form the MD [28]. During embryogenesis, the coelom itself derives from the lateral plate mesoderm that emanates from the primitive streak [33]. Similar to ontological development, our gene expression analysis indicated that nMUM first initiated mesendoderm differentiation and then influenced mesodermal lineage commitment in the differentiating human EBs. Rather than simply acting as an external cue to initiate EB self-organisation [34], our results suggest nMUM created morphogen gradients that influenced lineage commitment of differentiating hESCs, similar to other co-culture experiments [35]. Our previous microarray analysis in the nMUM (unpublished data) showed gene expression of several members of the TGF-beta super family of ligands (BMP, Activin) and Wnt family ligands consistent with other reports [36], [37], [38]. We propose that these and other morphogens in the nMUM initiated formation of mesendodermal cells and their subsequent differentiation in the EB. Further investigations are required to unravel the exact combination of mesenchymal soluble factors responsible for this induction. Following in vitro mesodermal lineage commitment, subsequent in vivo development of the hESC derived FRT epithelium at earlier time points mimicked known stages of MD development [27], both in morphological and molecular features previously described for human and mouse MD [24], [28].

A persistent problem in developing both in vitro and in vivo human organogenesis models from hESCs is the heterogeneity of tissue types produced. Similar to recent tissue recombination studies involving pluripotent cells [4], [5], [10], our method employing organ specific mesenchyme for generation of human FRT epithelium also generated a large fraction of ‘off target’ cell types. Although, manipulating the ratio of mesenchyme and hESCs could enhance differentiation and reduce unwanted lineage differentiation [4], a more consistent and robust outcome might be achieved if the appropriate inductive growth factors could be identified. In this study, we combined both techniques by first pre-differentiating EBs in the presence of mesendoderm promoting factors and then recombining them with inductive mesenchyme. Future studies aimed at identifying mesenchymally derived inductive factors will enable further refinement and control over the direction and efficiency of differentiation.

Overall, we demonstrated for the first time that nMUM can direct differentiation of hESCs to form human FRT epithelium. The entire process can be dissected and studied over a period of 8 weeks (Figure 5), providing an avenue to investigate human MD development. Both host environment and mesenchyme can be manipulated to examine systemic and local factors involved in MD development and maturation. Furthermore, our model of human FRT development may also provide a platform for the characterisation of human endometrial stem/progenitor cells recently identified in human and mouse uterus [1], [2], [39]. Indirect evidence suggests that stem/progenitor cells reside in the basalis (non-shedding layer) of the endometrium and it is postulated that they are remnants of fetal MD epithelium [40]. However, the isolation and identification these cells have been hampered by lack of defining cell surface markers. In this context, the availability of hESC derived fetal human MD epithelium may facilitate the search for cell surface markers that can be used to locate rare adult stem cells residing in the adult human uterus. Identifying these cells has the potential to profoundly impact on understanding normal physiological processes and diseases affecting the female reproductive tract epithelium, such as endometriosis, endometrial hyperplasia and endometrial cancer [41], [42].

## Materials and Methods

### Animals

Animals were obtained from Monash Animal Services. Day 1 nMUM and epithelium were obtained from female C57BL/6JAsmu (F1) mice. Female NOD.CB17-prkdcscid/Asmu (NOD.Scid) and NOD/Scid II-2R Gamma (NSG) mice 4–6 weeks old were housed under controlled environmental conditions at 20°C with 12-hour dark/light cycles and unlimited access to food and water. All animal handling and procedures were carried out in accordance with National Health and Medical Research Council of Australia guidelines for the Care and Use of Laboratory Animal Act and approval was obtained from the Monash University Animal Ethics Committee at Monash Medical Centre – A (MMC-A), Clayton, Australia. The approval numbers are 2006/44 and 2010/41.

### hESC Lines and Tissue Recombination

Two genetically modified hESC lines were used; the constitutively GFP^+^ line, ENVY [18] and MIXL1^GFP/wt^ reporter line [17], both of which were derived from HES3 (karyotype 46, XX) [43]. For differentiation experiments, 3000 hESCs per well were aggregated by centrifugation to form spin EBs in serum-free BPEL medium according to previously established protocols [44], [45]. In some experiments, the medium was supplemented with 50 ng.ml^−1^ BMP4 and 20 ng.ml^−1^ ACTIVIN A (both from R&D Systems). The uterine tubes of postnatal day 1 mice were mechanically dissected following enzymatic treatment as described in previous studies [7], [46]. Two small pieces of epithelial-free mesenchyme measuring 0.5 mm were recombined with day 1 EBs by co-culture in serum-free BPEL medium with or without growth factors (BMP4, 50 ng.ml^−1^, ACTIVIN A 20 ng.ml^−1^). On day 3 or 5, recombinants were re-suspended in 10 µl of neutralised rat tail collagen (a gift from Dr. Renea Taylor, Anatomy and Developmental Biology, Monash University, Australia) and placed on the surface of non-adherent Petri dishes for 10–15 minutes at 37°C to allow the gel to solidify. Medium was then added to the Petri dish and recombinants in collagen droplets were immersed in the medium and incubated at 37°C for 30 minutes prior to transplantation into NOD.Scid or NSG mice.

### Xenografting tissue recombinants

Tissue recombinants or mesenchyme alone and differentiated hESCs alone (EBs) were grafted under the renal capsule of female (5–6 weeks old) NOD.Scid or NSG mice for 2 to 8 weeks as previously described [5], [47]. All mice were ovariectomized at the time of surgery. For experiments assessing the functional capacity of hESCs-derived reproductive tract epithelium, animals were subjected to hormonal treatment one week before harvesting. Hosts carrying tissue recombinants were injected daily with 500 ng estradiol valerate (E2) in 100 µl corn oil (experiment group) or corn oil alone (control group) for 5 days. All grafts were harvested and fixed overnight in 4% paraformaldehyde (PFA), imaged, paraffin embedded and 3 µm serial sections cut for histology and immunohistochemistry. Some recombinants were harvested on day 5 following in vitro incubation for histology. These grafts were first re-suspended in collagen and fixed with 4% PFA prior to processing.

### Real time PCR

Individual recombinants and EBs were harvested from in vitro culture for gene expression analysis on days 3, 5, and 7, and 9. Samples were incubated in TrypLE Select (Invitrogen) for 30 minutes at 37°C then disaggregated by mixing, and digested completely with Lysis Solution provided with the RNA extraction kit (Ambion, Applied Biosystems, CA,). Following RNA extraction, DNase treatment was performed to eliminate contaminating genomic DNA using RNaqueous Micro DNase Treatment Kit (Ambion, Applied Biosystems). The quality and quantity of the RNA was checked using a Nanodrop Spectrophotometer ND-1000 in conjunction with ND-1000 V3.3.1 computer software (Thermofisher Scientific). Approximately 50 ng of total RNA from each sample was reverse transcribed to first strand cDNA with random hexamer primers using Superscript III reagents with RNase Inhibitor (Invitrogen). Real-time PCR was performed using Taqman gene expression probes, Taqman reagents and the 7900 HT Fast Real-Time PCR system absolute thermal cycler with software from Applied Biosystems. PCR reactions for all samples were run in triplicates. The comparative cycle threshold (Ct) method was used to analyse data. Gene expression levels were compared to the reference gene (REF), glyceraldehye-3-phosphate dehydrogenase (GAPDH). Since gene expression is inversely proportional to the Ct, the expression for target gene relative to GAPDH was calculated according to previously described formula below [48], [49].
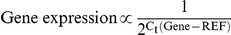
For purposes of presentation, we multiplied calculated values normalised to GAPDH by 1,000.

We tested for species cross reactivity of individual genes from information available on manufacturer's website; we performed a BLAST search using the amplicon to determine the gene's location in the human genome (GenBank mRNA reference numbers) against the mouse genome. The species specificity of each assay was tested by including neonatal uterine mesenchyme and human tissue in real time PCR reactions (data not shown). The inventoried Taqman Gene Expression Assays (Applied Biosystems) used in this study were: *GAPDH*: Hs99999905_m1, *MIXL1*: Hs00430824_g1, *BRACHYURY:* Hs00610080_m1, *GOOSECOID*: Hs00418279_m1, *OSR1*: Hs00377071_m1, *PAX2:* Hs01057415_m1, *POU5F1 (OCT4)*: Hs03005111_g1. The ID suffix “_m” indicates the probe spans an exon junction and will not detect genomic DNA. “_g” indicates an assay that may detect genomic DNA. The assay primers and probes may also be within a single exon. While genomic DNA amplification was possible for *MIXL1* and *POU5F1*, this was highly unlikely because of the DNase digestion step after RNA purification.

### Immunohistochemistry/Immunofluorescence

Sections were deparaffinized and hydrated through xylene and graded alcohols. Antigen retrieval was performed by microwaving sections in 0.01 M citrate buffer solution, pH-6 for 20 min, followed by cooling to room temperature. Sections stained with antibodies specific for nuclear proteins were permeablized with 0.5% Triton-X 100 in PBS for 10 minutes. Endogenous peroxidase activity was blocked with 3% H_2_O_2_ in methanol for 10 minutes at room temperature followed by 3 rinses with PBS. Protein Block was applied to minimise non-specific antibody binding (Serum-free protein block, DAKO, Denmark) for 10 minutes at room temperature. Concentration matched mouse IgG isotype negative controls were included in each run, rabbit IgG (DAKO) and goat IgG (Santa Cruz) were included in runs using polyclonal antibodies as negative controls. Following overnight incubation with primary antibodies at 4°C in a humidified chamber, slides were washed with PBS, and incubated with reagents from appropriate kits or stained with Alexa Fluor secondary antibodies (Table 1) at 1∶200, RT for 30 minutes; Mouse or Rabbit ENVISION (DAKO), LSAB HRP for Mouse/Rabbit/Goat (DAKO) were used according to manufacturer's protocols. Immunoreactivity was detected by incubating with DAB (DAKO) for 10–15 minutes, then rinsing with distilled water, followed by haematoxylin counterstain. Refer to (Table 1) for a list of primary antibodies used in this study. Hoechst 33258 staining was performed for 30 seconds (Molecular Probes, Eugene, Oregon, USA). Bright field images were taken with an Olympus upright microscope (Olympus Corporation, Tokyo, Japan). Fluorescent images were taken with a Leica DMR upright fluorescence microscope (Leica Microsystems, Mannheim, Germany). Individual color images were merged using Image J analysis software. Adult human endometrial sections were included as positive controls for every staining run.

**Table 1:** Primary antibody used in this study.

Primary Antibody	Species	Clone	Dilution	Source	Reference
α-SMA^4^	Mouse	1A4	1∶100	Dako	[1]
Anti-GFP^1^	Mouse	N/A	1∶500	Millipore	[50]
Anti-GFP^2^	Rabbit	N/A	1∶500	Abcam	
Anti-β –Tubulin*	Mouse	TUB2.1	1∶1000	Sigma-Aldrich	[51]
CA125^1^	Mouse	OC125	1∶200	Invitrogen	[12]
CK18^1^	Mouse	DC10	1∶500	Dako	[52]
ERα^1^	Mouse	6F11	1∶50	Nova Castra	[53]
Glycodelin A^4^	Mouse	001-13	1∶100	Abcam	[54]
HOXA10^3^	Goat	N/A	1∶1000	Santa Cruz	[55]
Ki67^4^	Rabbit	SP6	1∶200	ThermoScientific	[56]
PAX2^1^	Rabbit	N/A	1∶100	Invitrogen	[57], [58]
Vimentin*	Mouse	V9	1∶500	Sigma-Aldrich	[1]

1 **secondary antibody:** goat anti-mouse IgG_1_-AlexaFluor 488 (Invitrogen) or goat anti-mouse IgG_1_-AlexaFluor 568.

2 **secondary antibody:** donkey anti-rabbit AF 488 or donkey anti-rabbit AF 568 (Invitrogen).

3 **secondary antibody:** donkey anti-goat AF568 (Invitrogen).

4 **HRP kits**; mouse or rabbit envision (Dako) depending on the host species of the primary antibody.

*primary antibody is CY3 conjugated.

### 3D modelling of harvested tissue recombinant

Consecutive H&E and immunohistochemically stained serial sections of the tissue were imaged in their entirety using the Olympus DotSlide system (Olympus BX51, Olympus Corporation, Tokyo, Japan). The system utilised a 10× 0.3NA objective to image multiple connected fields-of-view and digitally joined the images together to form a single image covering the entire tissue section. The images of consecutive serial sections were then stacked into a 3D volume and aligned using AutoAligner v6.0.1 (Bitplane AG). The 3D tissue volume was then visualised and analysed using Imaris v7.0 (Bitplane AG).

## Supporting Information

Figure S1 **Xenograft size and hESC-derived FRT epithelium orientation.** (**A**) Size of grafts derived from EBs alone, growth factor (BMP4 and ACTIVIN A) treated EBs alone, recombinants and growth factor treated recombinants after 8 weeks in vivo incubation. Inset shows mean volume of three groups; GF EB, GF Recomb, Recomb, data plotted as mean ± s.e.m. (n = 4 per group, except for n = 2 for recombinant group). (**B**) H&E of a section from week 8 recombinant graft showing hESC derived epithelium grown in proximity to other epithelial and connective tissue structures (arrows indicate transplanted mouse stromal cells) (**C**) Composite image of a GF recombinant graft. The Hoechst stain shows hESC derived FRT epithelium structure comprising human epithelial cells with smooth nuclei surrounded by mouse stromal cells with speckled nuclei (arrows). All images were captured on ×4 magnification, inset was captured on ×40. (**D**) H&E of a section from week 4 recombinant graft showing two hESC derived epithelium in the same field of view (arrows indicate transplanted mouse stromal cells). Abbreviations: C, cartilage; E, epithelium; FC, Fluid-filled cavity; GF, growth factor; K, kidney; R, hESC derived FRT epithelium; S, stroma.
(TIF)

Figure S2 **Histogram summarising the percentage of grafts that contained hESC derived FRT epithelium, asterix indicates that week 2 & 4 grafts are included.**
(TIF)

Figure S3 **(A–B) representative ENVY hESC recombinant graft in vitro (C) representative H&E section of ENVY hESC recombinant graft consisting cells with distinct morphologies, two populations; ENVY hESC and nMUM.** Abbreviations: EB, embryoid body; M, neonatal mouse uterine mesenchyme.
(TIF)

Figure S4 **(A–B) representative section demonstrating ERα expression in hESC derived epithelium in grafts with (A) or without (B) E2 treatment (arrows indicating weakly stained nuclei), full arrows in (A) indicate mouse uterine stromal cells (C, D) representative sections showing ERα expression in normal human adult proliferative uterine glands and stroma.** Abbreviations: E, epithelium; S, stroma.
(TIF)

Figure S5 **Immunofluorescent images showing co-localisation of GFP^+^ hESC derived epithelium from 4 week grafts with (A) HOXA10 (arrows indicate nuclear staining), (B) PAX2 (arrows indicating partial/diffuse nuclear staining), (C) VIMENTIN, and (D) CA125 (arrows on cell surface).**
(TIF)

Figure S6 **(A–D) are constituents of the composite images in** **Figure 1C–F** **respectively.** DAPI stained images in (**E**) and (**F**) are serial sections corresponding to glandular structures depicted in Figure **1I** and **1K** respectively illustrating that hESC derived epithelium (smooth nuclei) is surrounded by mouse stromal cells (arrows, speckled nuclei). Abbreviations: CA125, Cancer Antigen 125; E, epithelium; HOX, Homeobox A10; PAX2, Pair box gene 2; S, stroma; Vim, VIMENTIN.
(TIF)
