# Supplementary figures and images for: Generation of Human Female Reproductive Tract Epithelium from Human Embryonic Stem Cells
Source: PLoS One. 2011 Jun 15;6(6):e21136. doi: 10.1371/journal.pone.0021136 (PMC3115988; doi:10.1371/journal.pone.0021136)

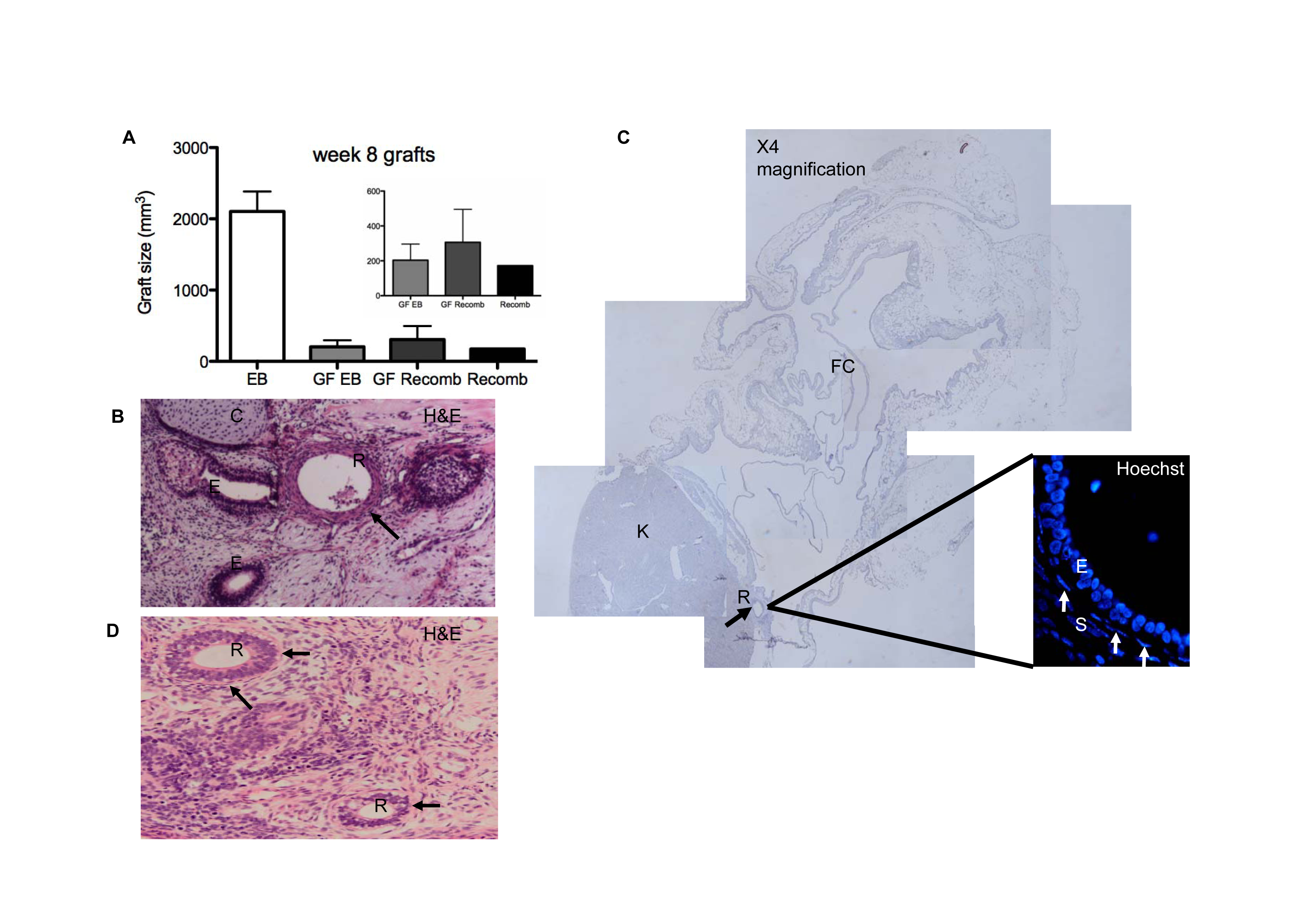

Supplement: Figure S1 — Xenograft size and hESC-derived FRT epithelium orientation. (A) Size of grafts derived from EBs alone, growth factor (BMP4 and ACTIVIN A) treated EBs alone, recombinants and growth factor treated recombinants after 8 weeks in vivo incubation. Inset shows mean volume of three groups; GF EB, GF Recomb, Recomb, data plotted as mean ± s.e.m. (n = 4 per group, except for n = 2 for recombinant group). (B) H&E of a section from week 8 recombinant graft showing hESC derived epithelium grown in proximity to other epithelial and connective tissue structures (arrows indicate transplanted mouse stromal cells) (C) Composite image of a GF recombinant graft. The Hoechst stain shows hESC derived FRT epithelium structure comprising human epithelial cells with smooth nuclei surrounded by mouse stromal cells with speckled nuclei (arrows). All images were captured on ×4 magnification, inset was captured on ×40. (D) H&E of a section from week 4 recombinant graft showing two hESC derived epithelium in the same field of view (arrows indicate transplanted mouse stromal cells). Abbreviations: C, cartilage; E, epithelium; FC, Fluid-filled cavity; GF, growth factor; K, kidney; R, hESC derived FRT epithelium; S, stroma. (TIF) [file pone.0021136.s001.tif]

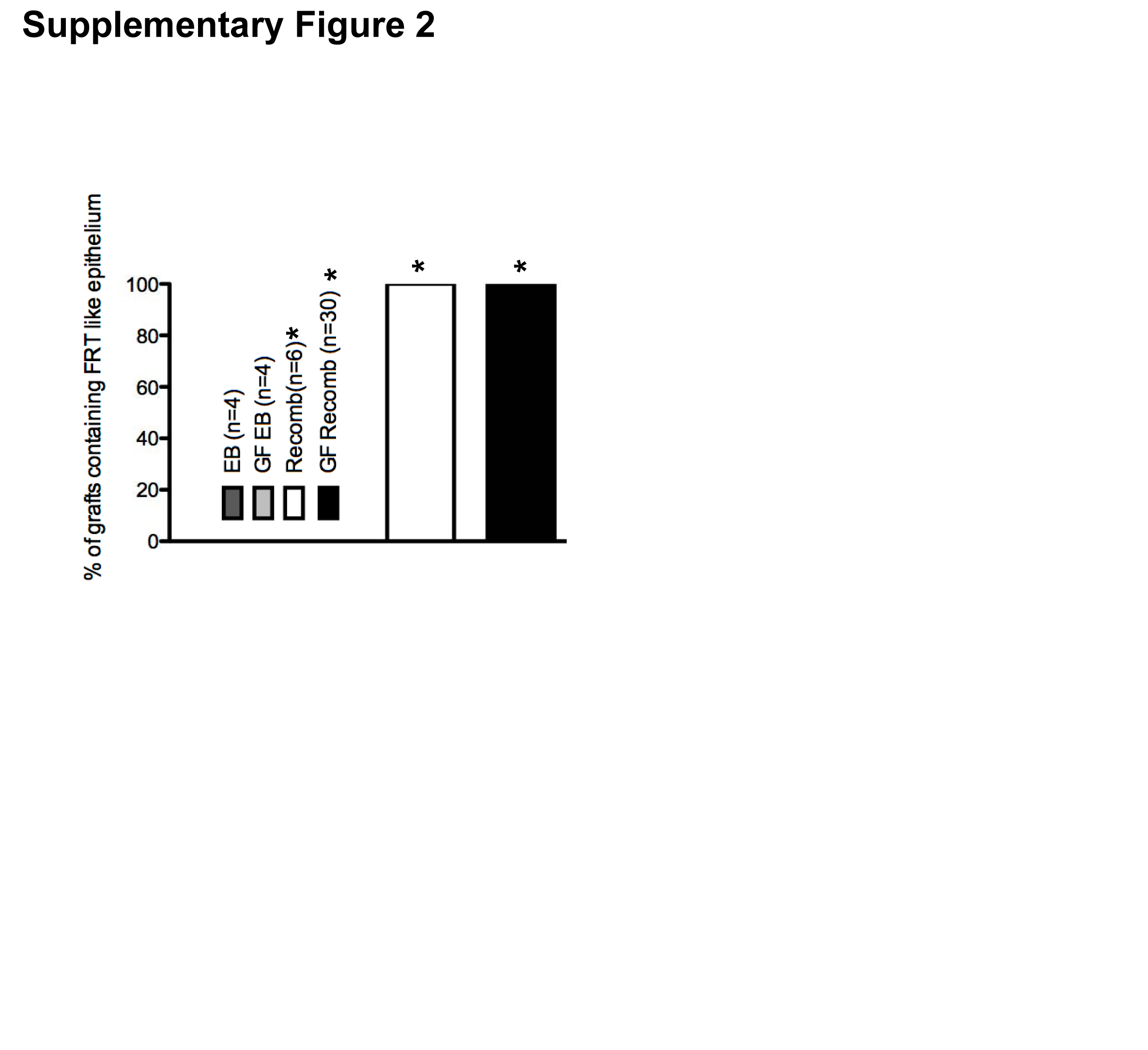

Supplement: Figure S2 — Histogram summarising the percentage of grafts that contained hESC derived FRT epithelium, asterix indicates that week 2 & 4 grafts are included. (TIF) [file pone.0021136.s002.tif]

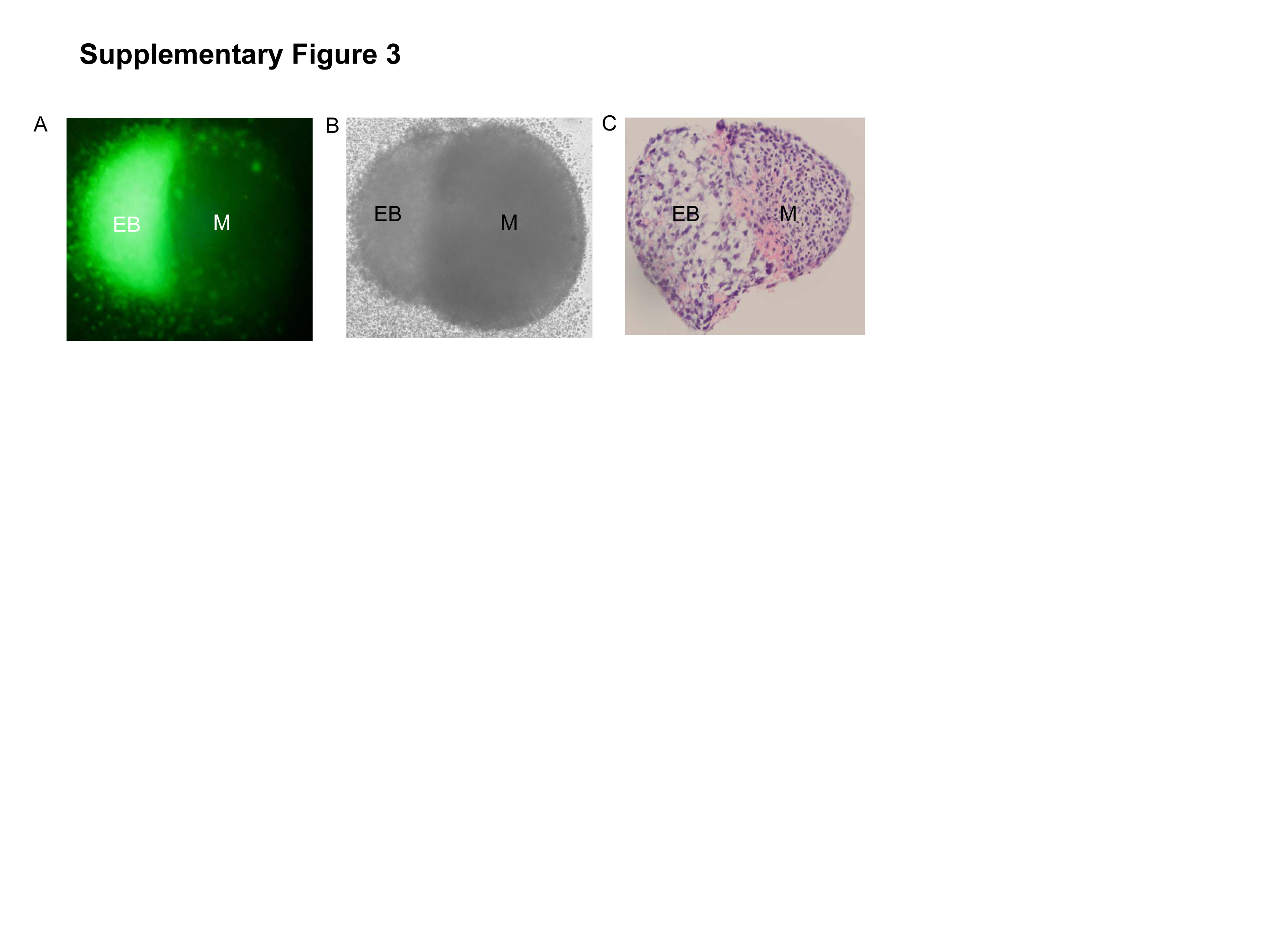

Supplement: Figure S3 — (A–B) representative ENVY hESC recombinant graft in vitro (C) representative H&E section of ENVY hESC recombinant graft consisting cells with distinct morphologies, two populations; ENVY hESC and nMUM. Abbreviations: EB, embryoid body; M, neonatal mouse uterine mesenchyme. (TIF) [file pone.0021136.s003.tif]

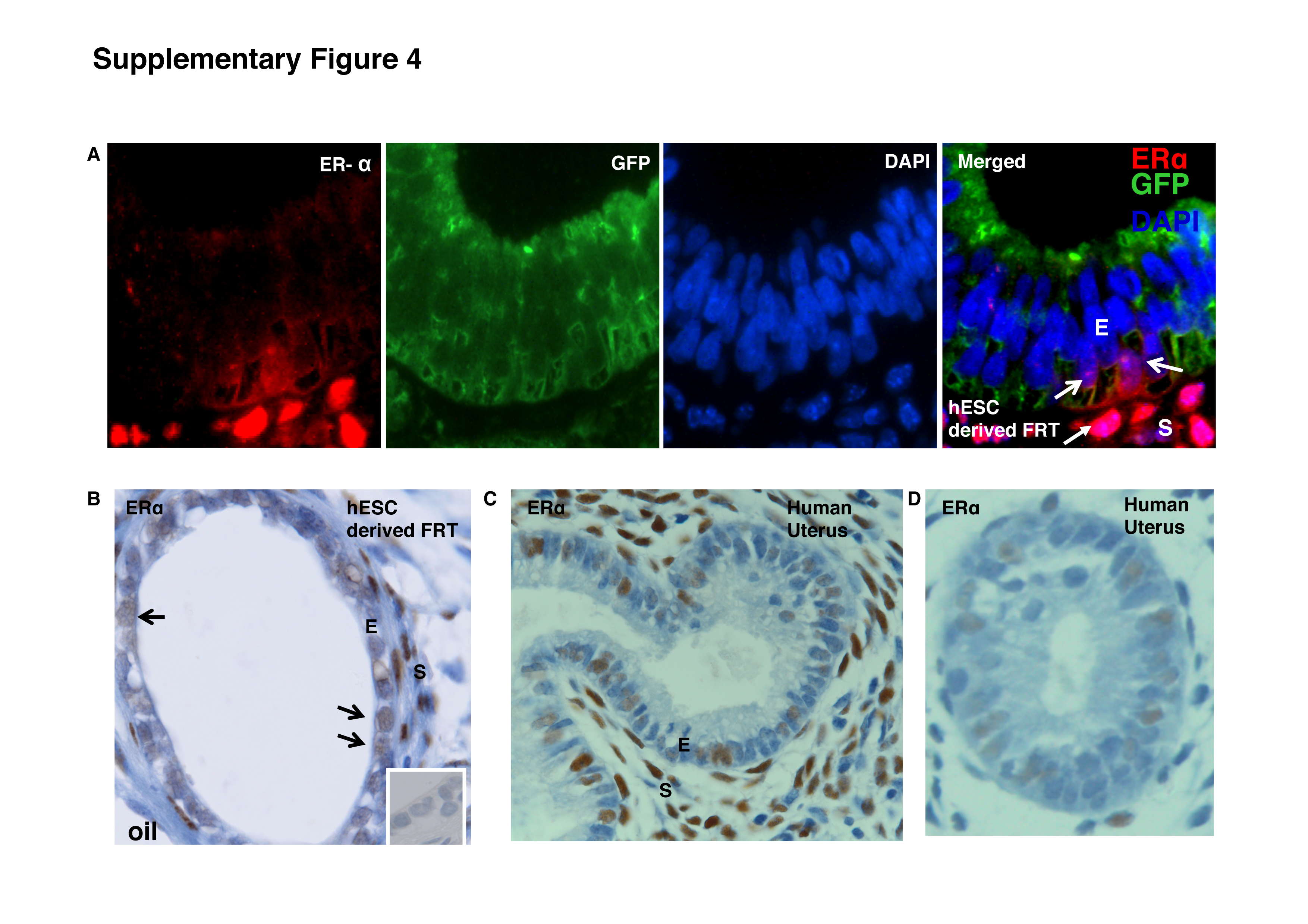

Supplement: Figure S4 — (A–B) representative section demonstrating ERα expression in hESC derived epithelium in grafts with (A) or without (B) E2 treatment (arrows indicating weakly stained nuclei), full arrows in (A) indicate mouse uterine stromal cells (C, D) representative sections showing ERα expression in normal human adult proliferative uterine glands and stroma. Abbreviations: E, epithelium; S, stroma. (TIF) [file pone.0021136.s004.tif]

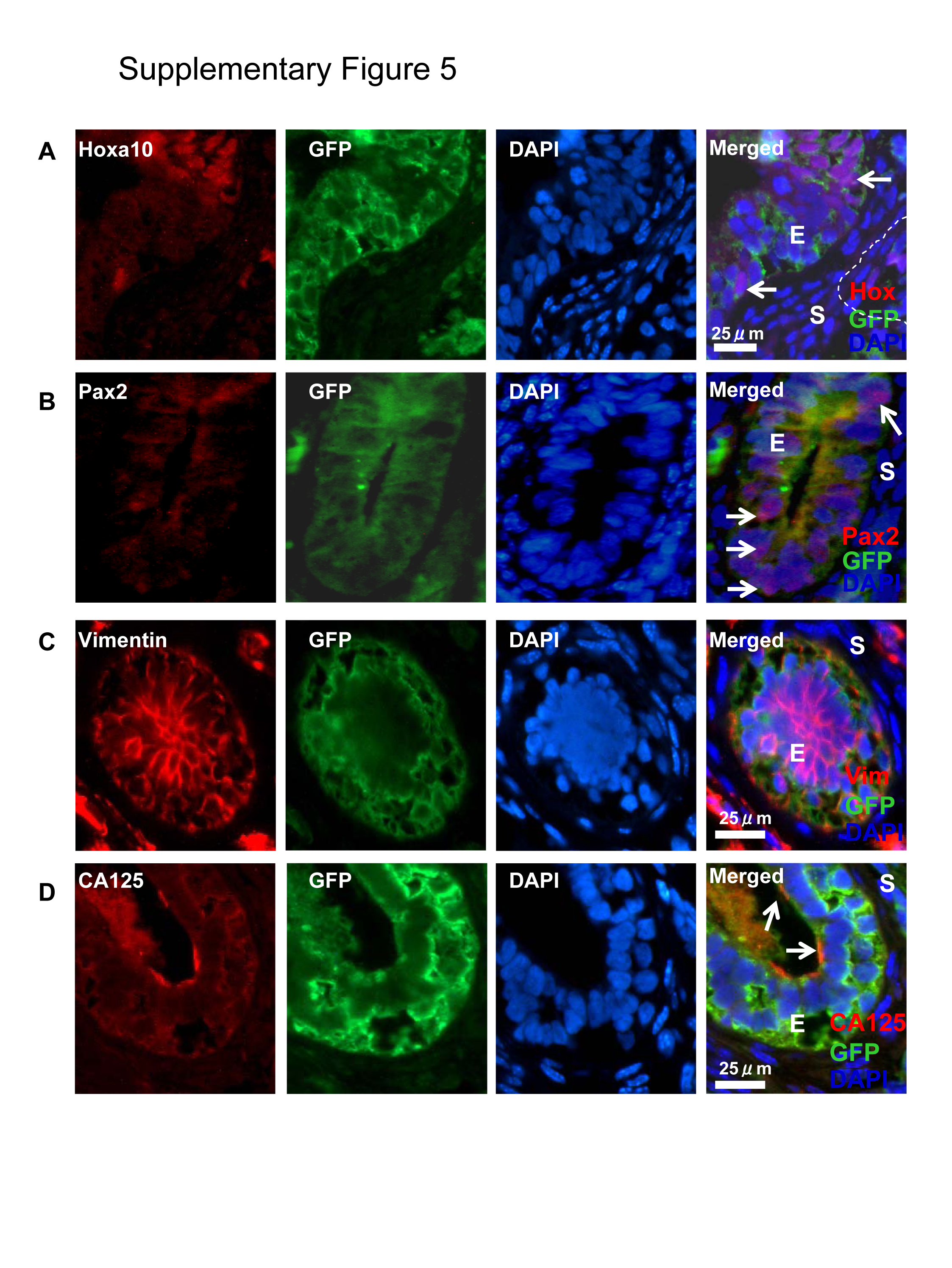

Supplement: Figure S5 — Immunofluorescent images showing co-localisation of GFP+ hESC derived epithelium from 4 week grafts with (A) HOXA10 (arrows indicate nuclear staining), (B) PAX2 (arrows indicating partial/diffuse nuclear staining), (C) VIMENTIN, and (D) CA125 (arrows on cell surface). (TIF) [file pone.0021136.s005.tif]

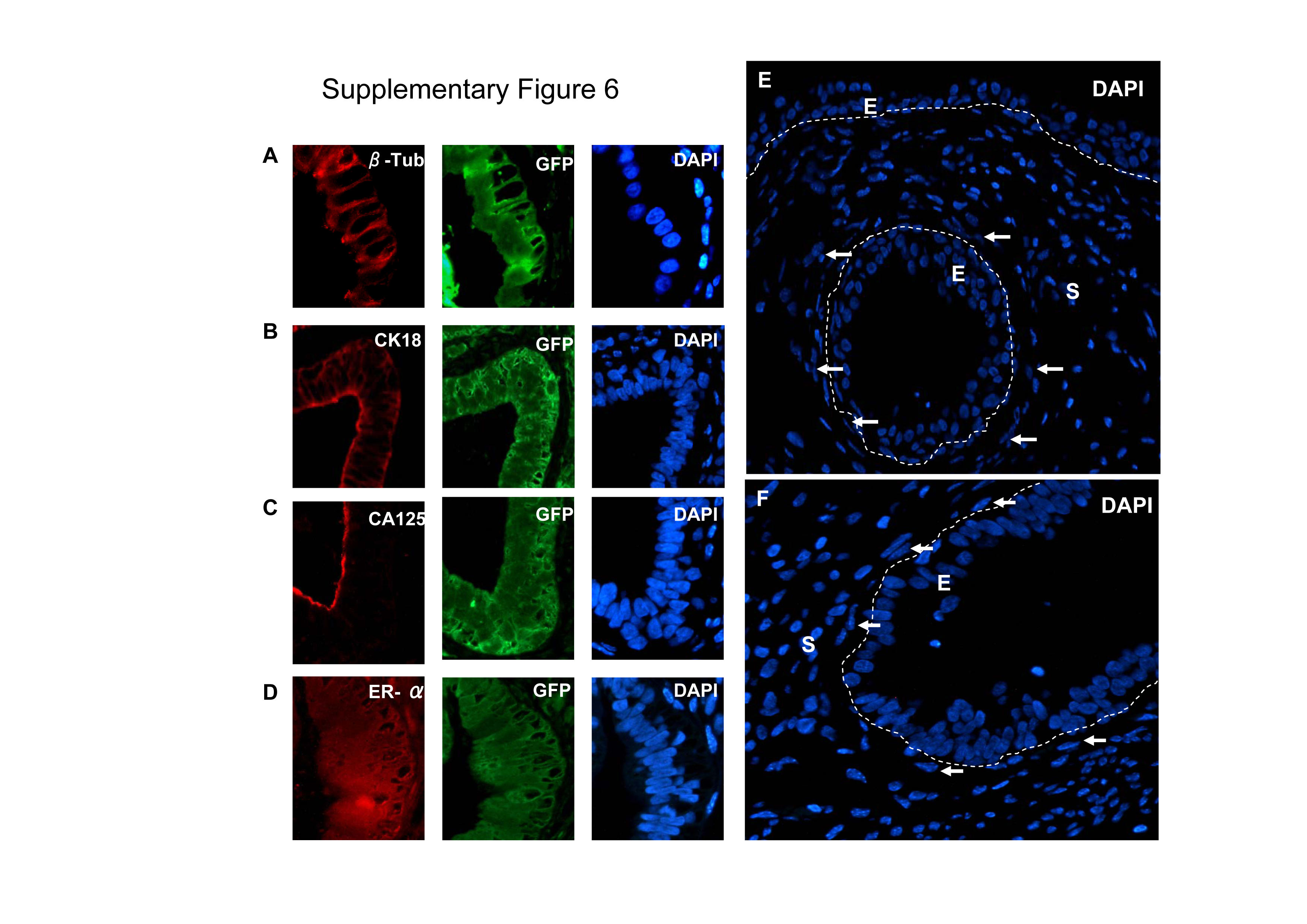

Supplement: Figure S6 — (A–D) are constituents of the composite images in Figure 1C–F respectively. DAPI stained images in (E) and (F) are serial sections corresponding to glandular structures depicted in Figure 1I and 1K respectively illustrating that hESC derived epithelium (smooth nuclei) is surrounded by mouse stromal cells (arrows, speckled nuclei). Abbreviations: CA125, Cancer Antigen 125; E, epithelium; HOX, Homeobox A10; PAX2, Pair box gene 2; S, stroma; Vim, VIMENTIN. (TIF) [file pone.0021136.s006.tif]
